# Everolimus plus reduced calcineurin inhibitor prevents *de novo* anti-HLA antibodies and humoral rejection in kidney transplant recipients: 12-month results from the ATHENA study

**DOI:** 10.3389/frtra.2023.1264903

**Published:** 2023-10-27

**Authors:** Wolfgang Arns, Aurélie Philippe, Vanessa Ditt, Ingeborg A. Hauser, Friedrich Thaiss, Claudia Sommerer, Barbara Suwelack, Duska Dragun, Jan Hillen, Christiane Schiedel, Anja Elsässer, Björn Nashan

**Affiliations:** ^1^ Department of Nephrology and Transplantation, Cologne Merheim Medical Center, Cologne, Germany; ^2^ BIH Biomedical Innovation Academy, Berlin Institute of Health at Charité—Universitätsmedizin Berlin, Berlin, Germany; ^3^ Department of Nephrology and Medical Intensive Care, Charité—Universitätsmedizin Berlin, Corporate Member of Freie Universität Berlin and Humboldt-Universität zu Berlin, Berlin, Germany; ^4^ Institute of Transfusion Medicine, Kliniken der Stadt Köln, Cologne, Germany; ^5^ Department of Nephrology, Goethe-University Frankfurt, Frankfurt, Germany; ^6^ III. Department of Medicine, University Medical Center Hamburg-Eppendorf, Hamburg, Germany; ^7^ Nephrology Unit, University Hospital Heidelberg, Heidelberg, Germany; ^8^ Department of Internal Medicine, Transplant Nephrology, University Hospital of Münster, Münster, Germany; ^9^ Department of Nephrology and Intensive Care Medicine, Charité Universitätsmedizin Berlin, Berlin, Germany; ^10^ Department of Immunology, Novartis Pharma GmbH, Nürnberg, Germany; ^11^ Department of Hepatobiliary Surgery and Transplantation, University Medical Center Hamburg-Eppendorf, Hamburg, Germany; ^12^ Organ Transplantation Center, Anhui Provincial Hospital, The First Affiliated Hospital of University of Science and Technology of China, Hefei, China

**Keywords:** everolimus, HLA antibodies, kidney transplant, DSA, reduced calcineurin inhibitor

## Abstract

**Background:**

Studies prospectively monitoring *de novo* donor-specific antibodies (dnDSAs) and their clinical impact are sparse. This substudy of ATHENA was initiated to evaluate the effect of everolimus (EVR) or mycophenolic acid (MPA) in combination with reduced calcineurin inhibitor (CNI, tacrolimus [TAC] or cyclosporine [CsA]) on the formation of human leukocyte antibodies (HLA), including dnDSA, and the impact on clinical outcomes in kidney transplant (KTx) recipients.

**Methods:**

All eligible patients were randomized 1:1:1 to receive either EVR + TAC, EVR + CsA or MPA + TAC, with basiliximab induction plus steroids after transplantation up to Month 12. The incidence of dnDSA by treatment group and the association with clinical events were evaluated descriptively as an exploratory objective in the intent-to-treat (ITT) and per-protocol (PP) populations with at least one antibody assessment.

**Results:**

Overall, none of the patients in the EVR + TAC group had either dnDSA or antibody mediated rejection (PP or ITT population) and only one patient with dnDSA in the TAC + MPA group had antibody mediated rejection.

**Conclusion:**

The EVR regimen was comparable to MPA regimen with an extremely low incidence of dnDSA over 1 year of treatment.

## 1. Introduction

The development of *de novo* donor-specific human leukocyte antigen (HLA) antibodies (dnDSA) is a major risk factor for acute and chronic antibody-mediated rejection (AMR) and graft loss after kidney transplantation (KTx) (1). Single center studies report that acute AMR was more common in KTx recipients with dnDSA vs. without dnDSA (18.8% vs. 0%, respectively; *P* < 0.001) (2) and probability of graft survival was lower (79% vs. 94%, respectively; *P* = 0.05) (3). In a case-control study, dnDSA were more common in patients with graft failure vs. controls without graft failure (54% vs. 16%, respectively; *P* < 0.001), and chronic active AMR was significantly more common in patients with dnDSA compared to those without dnDSA (61% vs. 12%, *P* < 0.001) (4). The prevalence of dnDSAs is generally between 5% and 10% at 1-year post KTx and slowly increases thereafter to 20% at 5 years (5). Risk factors for dnDSA development include a high number of HLA mismatches (especially DQ mismatches), and inadequate immunosuppression and non-adherence (1). Since dnDSA development has been associated with worse outcomes, it is important to avoid this undesirable alloimmune response and understand the effects of different immunosuppressive agent combinations on dnDSA formation. Although B cells and plasma cells produce antibodies, T cells also play role in the development of dnDSA, and effective T-cell suppression is required to prevent dnDSA formation (6).

Everolimus (EVR), an inhibitor of mammalian target of rapamycin (mTORi), permits reduced calcineurin inhibitor (CNI) exposure after KTx (7). CNIs affect humoral immune response by acting on T cells, whereas mTORis, such as EVR, affect both T cells and B cells (6). Previous studies with CNI-free EVR-based regimens in KTx patients demonstrated increased formation of dnDSA and transplant rejection compared with CNI (8–10). Also, Liefeldt and colleagues reported an increased risk for dnDSA after conversion to CNI-free therapy with EVR, though another reason could be that a high percentage of patients were also steroid-free, and CNI- plus steroid-free regimen may have led to rejection (11). These CNI elimination immunosuppression regimens may result in inadequate immunosuppression, possibly contributing to DSA development and subsequent graft failure.

An alternative strategy using EVR with reduced CNI exposure was investigated in the ATHENA study, a large, randomized trial involving 612 *de novo* kidney transplant recipients (ClinicalTrials.gov identifier: NCT01843348; EudraCT number: 2011–005238–21) (7). ATHENA compared EVR + tacrolimus (TAC) or EVR + cyclosporine A (CsA) vs. a standard-of-care regimen of mycophenolic acid (MPA) + TAC; all patients received steroids. The 12-month results of the ATHENA study revealed a comparable efficacy of EVR + TAC or EVR + CsA to MPA + TAC and though non-inferiority of renal function with EVR + TAC/CsA was not achieved, an increase in renal function from Month 1 to 12 was comparable in both EVR and MPA groups (7). Given the paucity of data with regards to the development of dnDSA with EVR + reduced CNI-therapy in *de novo* kidney transplant recipients, this substudy of ATHENA was initiated to evaluate the effect of EVR or MPA in combination with reduced CNI on the formation of HLA antibodies, including dnDSA, and the impact on clinical outcomes over 1 year.

## 2. Material and methods

### 2.1. Study design and population

ATHENA was a 12-month, prospective, multicenter, randomized, controlled, parallel-group, open-label study in *de novo* kidney transplant recipients (who received a first kidney transplant). The study was conducted from December 27, 2012 through March 23, 2016 in Germany and France. Details of the ATHENA study, including complete inclusion/exclusion criteria, the immunosuppression regimen, and patient stratification have been described previously (7).

In brief, patients with preformed HLA antibodies not directed against the donor and with <20% panel reactivity at the time of transplant were included in the study (12). Patients with a current panel reactive antibody level of >20% (within 4 months before enrollment) were excluded from the study.

Patients were randomized 1:1:1 to receive either, EVR + TAC, EVR + CsA, or MPA + TAC. EVR was maintained at target trough concentration of 3–8 ng/mL throughout the study period. The target trough concentration of TAC in the EVR + TAC and MPA + TAC arms was 4–8 ng/mL until the end of Month 2 and 3–5 ng/mL thereafter. In the EVR + CsA arm, the target trough concentration of CsA was 75–125 ng/mL until the end of Month 2 and 50–100 ng/mL thereafter. MPA was used either as enteric coated mycophenolate sodium (1.44 g/day) or mycophenolate mofetil (2 g/day). All patients received basiliximab 20-mg induction therapy on Day 0 and 4, and steroids (≥5 mg/day) until Month 12 (Supplementary Figure S1). The study was conducted in accordance with the Declaration of Helsinki and the International Conference on Harmonization Guidelines for Good Clinical Practice. The study was approved by all competent Ethics Committees and regulatory authorities. Informed consent was obtained by investigators from all patients enrolled into the study.

### 2.2. Study outcomes and assessments

The primary objective of the ATHENA study was to demonstrate non-inferiority of renal function [as assessed by estimated glomerular filtration rate (eGFR), Nankivell formula] with EVR + TAC and/or EVR + CsA vs. MPA + TAC at Month 12 after transplantation (7). This substudy was performed with the objective to evaluate the incidence of HLA antibodies, including DSA, by treatment group and the association with acute rejection within 1 year post *de novo* KTx (12). Blood samples (5 ml) for all patients were collected at baseline, Month 6, and Month 12. The presence and evolution of HLA antibodies and DSA antibodies in serum at baseline and Month 12 were evaluated using Luminex® LABScreenTM Single Antigen Bead assays (One Lambda, CA, USA) according to the manufacturer's instructions (13). This method used color-coded microbeads coated with purified HLA-A, -B, -C, -DR and -DQ, and major-histocompatibility-complex (MHC) class I–related chain A (MICA) antigens. The beads were analyzed using Luminex xMAP multiplex technology. In principle, the presence of HLA antibodies was detected using a goat anti-human IgG coupled with phycoerythrin, and fluorescence of each bead was detected by the reader and recorded as mean fluorescent intensity (MFI) as described previously by our group (14). A cut-off of 500 MFI was selected based on the literature (15).

### 2.3. Statistical analysis

Patients who received at least one dose of the study drug were considered in the intent-to-treat (ITT) population and all ITT patients without any major protocol deviation were considered in the per-protocol (PP) population. This *post hoc* analysis included a statistical analysis plan that was developed after the database lock for analyses of preformed HLA antibodies and dnDSA. The analysis set included all transplanted patients with at least one antibody assessment (either HLA and/or non-HLA). The analysis of HLA antibodies was carried out descriptively. Demographic variables were analyzed using F-test for continuous variables and Fisher's exact test for categorical variables. The embedded subgroups of HLA antibodies were defined as preformed HLA: respective HLA class/loci detected at baseline (MFI ≥ 500) independent of mismatch; respective preformed critical HLA: HLA class/loci detected at baseline (MFI ≥ 500) although being a mismatch; and dnDSA: HLA class/loci detected: respective HLA class/loci increased from negative or <500 MFI at baseline to MFI ≥ 500 at endpoint and the HLA was specified as mismatch.

## 3. Results

### 3.1. Patient population

Of 655 patients randomized to treatment in ATHENA, 612 patients who received at least one dose of the study drug were included in the ITT population and 338 in the PP population (7). Of these, 606 (206 in EVR + TAC, 198 in EVR + CsA, and 202 in MPA + TAC) and 337 (110 in EVR + TAC, 80 in EVR + CsA, and 147 in MPA + TAC) patients, respectively, had at least one antibody assessment and were included in the ITT and PP analysis populations described herein for the HLA analyses. We limited the main analyses to the PP population since this population comprised patients who were on the assigned treatment regimen throughout the study without major protocol deviations and would better represent the effect of continuous immunosuppression. Results in the ITT population were comparable to those reported for the PP population and are presented in the Supplementary Tables S1–S3.

In general, baseline demographic and clinical characteristics of patients were well balanced between treatment groups except a numerically higher proportion of patients with 2 human leukocyte antigen-DR mismatches in the EVR + TAC and EVR + CsA groups compared with MPA + TAC group (Table 1 [PP population with HLA data] and Supplementary Table S1 [ITT population]). This finding was consistent to that reported for the ITT population in the primary ATHENA study (7).

**Table 1:** Baseline demographics and characteristics of patients in ATHENA HLA substudy cohort (PP population with HLA data at baseline).

Variable	EVR + TAC (*n* = 97)	EVR + CsA (*n* = 68)	MPA + TAC (*n* = 129)	Total (*N* = 294)	
Age					
Mean (SD), years	53.2 (12.2)	52.1 (12.0)	53.6 (11.4)	53.1 (11.8)	0.723^a^
≥65 years, *n* (%)	19 (19.6)	10 (14.7)	19 (14.7)	48 (16.3)	
Male, *n* (%)	69 (71.1)	49 (72.1)	84 (65.1)	202 (68.7)	0.533^b^
White, *n* (%)	91 (93.8)	67 (98.5)	126 (97.7)	284 (96.6)	0.265^b^
Mean BMI (SD), kg/m^2^	25.8 (3.6)	26.0 (3.9)	26.6 (4.3)	26.2 (4.0)	0.240^a^
Panel reactive antibodies, *n* (%)					
0	86 (95.6))	63 (96.9)	122 (96.1)	271 (96.1)	
≤10	3 (3.3)	1 (1.5)	5 (3.9)	9 (3.2)	
>10 and ≤20	1 (1.1)	0 (0.0)	0 (0.0)	1 (0.4)	
>20	0 (0.0)	1 (1.5)	0 (0.0)	1 (0.4)	
Missing	7	3	2	12	
Previous kidney transplant, *n* (%)	3 (3.1)	0 (0.0)	3 (2.3)	6 (2.0)	
HLA-A mismatches, *n* (%)					
0	25 (25.8))	15 (22.1)	42 (32.6)	82 (27.9)	
1	48 (49.5)	42 (61.8)	59 (45.7)	149 (50.7)	
2	24 (24.7)	11 (16.2)	28 (21.7)	63 (21.4)	
HLA-B mismatches, *n* (%)					
0	19 (19.6)	15 (22.1)	36 (27.9)	70 (23.8)	
1	46 (47.4)	27 (39.7)	44 (34.1)	117 (39.8)	
2	32 (33.0)	26 (38.2)	49 (38.0)	107 (36.4)	
HLA-DR mismatches, *n* (%)					
0	24 (24.7)	23 (33.8)	48 (37.2)	95 (32.3)	
1	53 (54.6)	33 (48.5)	66 (51.2)	152 (51.7)	
2	20 (20.6)	12 (17.6)	15 (11.6)	47 (16.0)	
Mean cold ischemia time (SD), h	10.8 (5.6)	11.7 (5.9)	10.8 (6.2)	11.0 (5.9)	
Participant in Eurotransplant Senior Program, *n* (%)	7 (7.2)	3 (4.4)	7 (5.4)	17 (5.8)	
Donor characteristics					
Mean age (SD), y	51.3 (15.8)	52.7 (14.0)	51.7 (14.4)	51.8 (14.7)	
Deceased heart-beating, *n* (%)	85 (87.6)	55 (80.9)	104 (80.6)	244 (83.0)	
Living-related, *n* (%)	7 (7.2)	9 (13.2)	16 (12.4)	32 (10.9)	
Living-unrelated, *n* (%)	5 (5.2)	4 (5.9)	9 (7.0)	18 (6.1)	

CsA, cyclosporine; EVR, everolimus; HLA, human leukocyte antigen; MPA, mycophenolic acid; PP, per-protocol; SD, standard deviation; TAC, tacrolimus.

^a^ F-test.

^b^ Fisher's exact test.

### 3.2. HLA antibodies

At baseline, HLA data were available for 294/337 patients in the PP population (97 in EVR + TAC, 68 in EVR + CsA, and 129 in MPA + TAC; Table 2). Data for 535/606 patients in the ITT population with HLA data available are shown in Supplementary Table S2. A total of 130 patients (44.2%) in the PP population had preformed HLA antibodies at baseline (EVR + TAC, 46.4%; EVR + CsA, 41.2%; and MPA + TAC, 44.2%; MFI ≥ 500 independent of mismatch) (Table 2).

**Table 2:** Patients in ATHENA HLA substudy cohort with preformed HLA and dnDSA antibody data (PP population).

Patient group	HLA data at BL, n	Preformed HLA at BL, *n* (%)^a^	Preformed critical HLA at BL, *n* (%)^a^	dnDSA, *n* (%)^b^
EVR + TAC (*N* = 110)	97	45 (46.4)	3 (3.1)	1 (1.0)
EVR + CsA (*N* = 80)	68	28 (41.2)	3 (4.4)	4 (5.9)
MPA + TAC (*N* = 147)	129	57 (44.2)	8 (6.2)	2 (1.6)
Total (*N* = 337)	294	130 (44.2)	14 (4.8)	7 (2.4)

Preformed HLA and preformed critical HLA at baseline and DSA detected at MFI ≥ 500.

BL, baseline; CsA, cyclosporine; dnDSA, *de novo* donor-specific antibodies; EVR, everolimus; HLA, human leukocyte antigen; MFI, mean fluorescent intensity; MPA, mycophenolic acid; PP, per-protocol; TAC, tacrolimus.

^a^ Percentage based on number of patients with HLA data at BL.

^b^ Percentage based on number of patients with HLA antibodies at BL and post-BL (*n* = 97 in EVR + TAC, 68 in EVR + CsA, and 125 in MPA + TAC groups; total *n* = 290).

**Figure 1:**
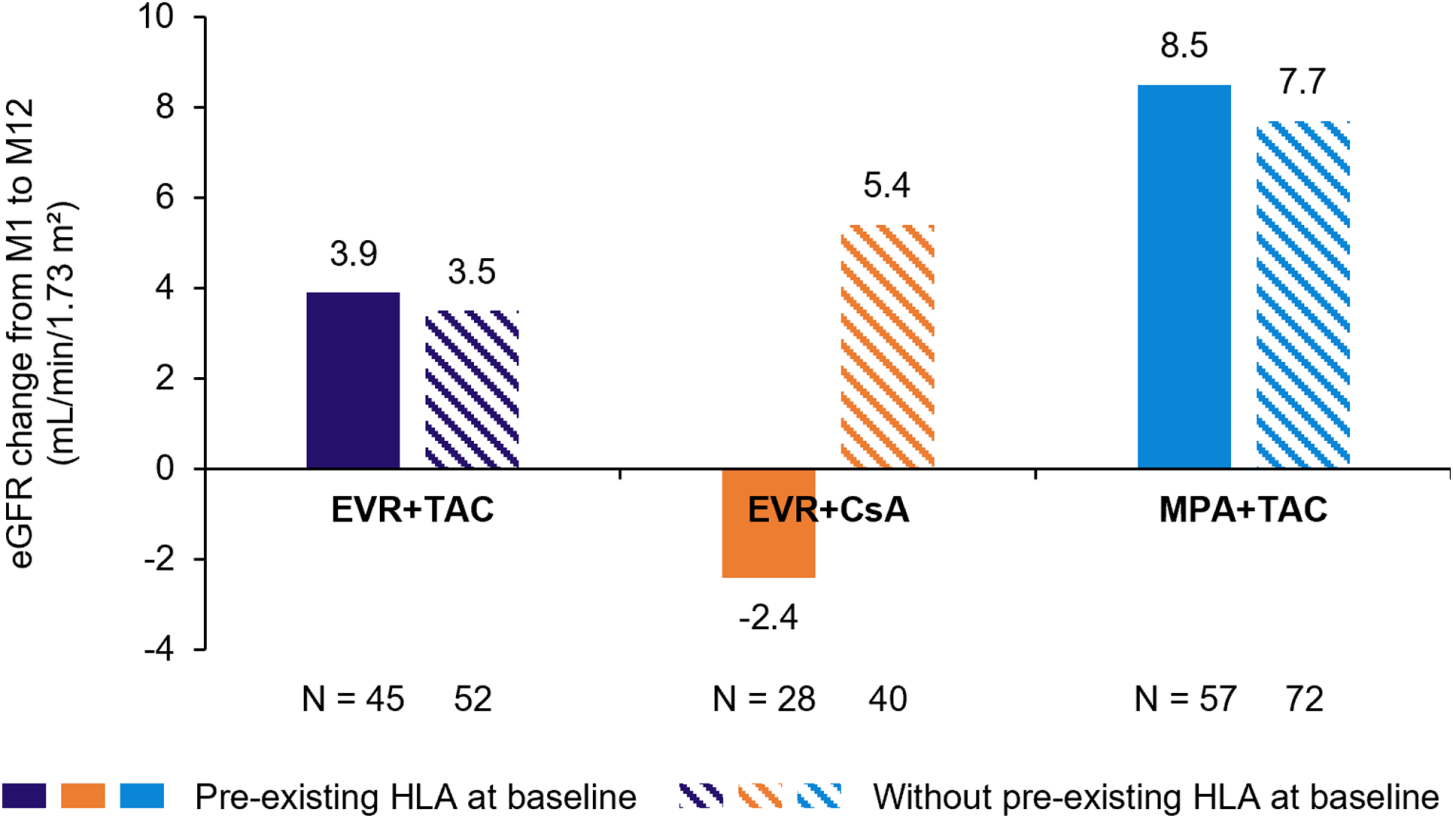
Change in renal function (eGFR, Nankivell) over 12 months in patients with vs. without preformed HLA antibodies (PP population). N is patients with eGFR data at Month 12. CsA, cyclosporine; eGFR, estimated glomerular filtration rate; EVR, everolimus; HLA, human leukocyte antigen; MPA, mycophenolic acid; PP, per-protocol; TAC, tacrolimus.

Overall, 8/130 (6.2%) patients from the PP population with preformed HLA had clinical events (EVR + TAC, 6.7%, EVR + CsA, 7.1%, and MPA + TAC, 5.3%) (Table 3). These clinical events were all biopsy-proven acute rejection (BPAR): 1 patient in the EVR + TAC group had evidence of AMR and the other 7 patients had no evidence of AMR. No patients experienced graft loss or death. Corresponding data for the ITT population are shown in Supplementary Table S3. In the PP population, the change in renal function (eGFR, Nankivell) from baseline to Month 12 was comparable between patients with or without preformed HLA at baseline treated with EVR + TAC (3.9 and 3.5 mL/min/1.73 m^2^, respectively) or MPA + TAC (8.5 and 7.7 mL/min/1.73 m^2^, respectively) (Figure 1).

**Table 3:** Clinical outcome of patients with preformed HLA and dnDSA (PP population).

	Clinical event	EVR + TAC (*N* = 110)	EVR + CsA (*N* = 80)	MPA + TAC (*N* = 147)	Total (*N* = 337)
Preformed HLA		*M* = 45	*M* = 28	*M* = 57	*M* = 130
	Overall, *n*/*M* (%)	3/45 (6.7)	2/28 (7.1)	3/57 (5.3)	8/130 (6.2)
	BPAR, *n*	3	2	3	8
	AMR, *n*	1	0	0	1
	Graft loss, *n*	0	0	0	0
	Death, *n*	0	0	0	0
Preformed critical HLA		*M* = 3	*M* = 3	*M* = 8	*M* = 14
	Overall, *n*/*M* (%)	0/3 (0)	0/3 (0)	0/8 (0)	0/14 (0)
	BPAR, *n*	0	0	0	0
	AMR, *n*	0	0	0	0
	Graft loss, *n*	0	0	0	0
	Death, *n*	0	0	0	0
dnDSA		*M* = 1	*M* = 4	*M* = 2	*M* = 7
	Overall, *n*/*M* (%)	0/1 (0)	1/4 (25.0)	1/2 (50.0)	2/7 (28.6)
	BPAR, *n*	0	1	1	2
	AMR, *n*	0	0	1	1
	Graft loss, *n*	0	0	0	0
	Death, *n*	0	0	0	0

Clinical event defined as biopsy-proven acute rejection, graft loss or death.

*M*, number of paitients with preformed/critical preformed/dnDSA at BL; *N*, total number of patients; n, number of patients with an event.

AMR, antibody-mediated rejection; BL, baseline; BPAR, biopsy-proven acute rejection; CsA, cyclosporine; dnDSA, *de novo* donor-specific human leukocyte antigen antibodies; EVR, everolimus; HLA, human leukocyte antigen; MPA, mycophenolic acid; PP, per-protocol; TAC, tacrolimus.

In the PP population, 14 patients had preformed critical HLA antibodies at baseline (MFI ≥ 500 although being a mismatch): EVR + TAC, 3.1%; EVR + CsA, 4.4%; and MPA + TAC, 6.2% (Table 2). None of these patients had a clinical event over Month 12 (BPAR, graft loss, or death; Table 3). Corresponding data for the ITT population are shown in Supplementary Table S3.

### 3.3. De novo donor-specific HLA antibodies

Across the 3 immunosuppressive regimens, only 7 patients in the PP population developed dnDSA (MFI ≥ 500) within 1 year after KTx. Similar rates of dnDSA development were observed in the EVR + CsA [1 patient (1%)] and MPA + TAC [2 patients (1.6%)] groups (Table 2). In the EVR + CsA group, 4 (5.9%) patients developed dnDSA. Of these 7 patients with dnDSA, 2 patients had clinical events: 1 patient in the EVR + CsA and MPA + TAC groups, respectively, both of whom experienced BPAR (Table 3). The patient in the EVR + CsA group had no evidence of AMR, whereas only 1 patient in the MPA + TAC group had evidence of AMR. No graft loss or death occurred in any patient with dnDSA in the PP population. Corresponding data for the ITT population is shown in Supplementary Table S3. Of note, neither in the PP nor in the ITT population dnDSA were observed in the EVR + TAC group.

## 4. Discussion

Primary results of the ATHENA study showed that EVR + reduced CNI was efficient and safe in *de novo* patients (7). The study showed that renal function was comparable between the EVR arms and standard-of-care regimen (MPA + TAC), and that immunosuppressive efficacy was similar in the EVR + TAC and MPA + TAC groups. In addition, both EVR-based arms had significantly fewer cytomegalovirus infections compared with the MPA + TAC regimen. This prospective substudy of ATHENA showed that EVR + reduced CNI was also very effective against formation of dnDSA. More importantly, after 12 months there was no substantial difference between the 3 immunosuppression regimens on the development of dnDSA, suggesting EVR does not increase the risk for dnDSA development.

Overall, the incidence of dnDSA was extremely low under conditions of a controlled clinical trial and dnDSA, at least in this setting, did not appear to adversely affect clinical outcomes. Our findings confirmed that AMR is not necessarily related to the development of dnDSA *per se*. Although the substudy was designed to investigate the impact of immunosuppressive regimens on the development of dnDSA, it was interesting to observe that the presence of preformed HLA antibodies (MFI ≥ 500 at baseline, independent of mismatch) and critical HLA antibodies (MFI ≥ 500 at baseline, although being a mismatch) had no influence on clinical outcome, irrespective of EVR or MPA exposure.

Previous preclinical studies involving kidney and heart allotransplantation models have demonstrated an inferior immunosuppressive ability with EVR vs. CNI exposure, indicating that CNI-free regimens present an immunological risk (16, 17). Also, clinical studies have suggested that CNI-free and CNI-sparing regimens may be associated with an increased risk of acute rejection (18, 19). Plus, in addition to acute rejection as an indicator of under-immunosuppression, the CNI-sparing regimen with EVR vs. MPA has been said to lead to the development of DSAs and AMR. Yet, on the contrary, other studies investigating CNI-minimizing regimens have not been associated with an increased incidence of acute rejection/immunological risk in *de novo* KTx patients (20–23), which goes well with the findings of this substudy. Here, we have presented an ideal platform to prospectively analyze the formation of DSAs in the ATHENA study and to determine if immunosuppression is adequate in controlling the emergence of DSAs and AMR.

In line with our findings, results from several randomized, controlled trials in *de novo* KTx patients have shown that EVR with reduced-exposure CNI is not associated with a higher incidence of dnDSA compared to standard-exposure CNI (24–26). Moreover, no difference in the incidence of acute rejection between groups was reported. In addition, our findings were comparable with another observational study in patients treated with MPA plus TAC, either once or twice daily over 2 years (incidence of DSA: 3.6% and 1.2% and AMR: 4.8% and 2.7%, respectively, with twice- and once-daily TAC) (27). Hence, these findings, in combination with our results, which both exhibited no increased risk for dnDSAs in patients receiving EVR with reduced CNI, strongly oppose results of other studies that suggest dnDSA may be more frequent in patients given EVR in a CNI-free regimen (8–10), likely reflecting inadequate immunosuppression with CNI-free regimens.

The role of induction therapy in preventing *de novo* DSA is also worth discussion. Studies have reported that induction with basiliximab is associated with lower incidence of *de novo* DSA in kidney transplant recipients compared with anti-depleting agent, alemtuzumab or rabbit antithymocyte globulin (rATG) (28). Bath NM et al. (2020) also reported that patients who received induction with basiliximab had lower incidence of *de novo* DSA than alemtuzumab, which could be because of elevated B-cell activating factor levels in patients treated with alemtuzumab (29). In our study, basiliximab was given as induction therapy in all treatment arms, all of which showed an extremely low incidence of DSA over 1 year treatment. Results from the TRANSFORM study in which majority of patients received induction with basiliximab (83%) showed comparable low incidence of *de novo* DSA on treatment with EVR + reduced CNI vs. MPA + standard CNI over 12 months (10.2% vs. 13.6%, *P* = 0.508) and 24 months (22.4% vs. 17.7%, *P* = 0.508) (24, 26). A retrospective analysis of a randomized trial reported comparable incidence of *de novo* DSA over 12 months in patients who received r-ATG + EVR + TAC [5/78 (6.4%)] vs. basiliximab + EVR + TAC [3/87 (3.4%)] vs. basiliximab + MPS + TAC [5/90 (5.5%)] (25). A multicenter analysis of 24 patients who were enrolled to 2-year, randomized phase-3 study (RAD001A1202 study) and treated with basiliximab + EVR + reduced CsA vs. basiliximab + MMF + standard CsA showed comparable rates of *de novo* DSA (15.4% vs. 18.3%) over 10 years (30).

The favorable effects observed in this study with the mTORi, EVR-based regimen, can be explained. The mTOR signaling plays an important role in the pathomechanism of HLA antibody-mediated endothelial cell activation and proliferation, which lead to rejection and vasculopathies. The HLA antibodies stimulate the activation of mTOR, as well as the downstream targets extracellular-signal regulated kinase (ERK), S6 kinase (S6K), and S6 ribosomal protein (S6RP) [the latter stimulating intercellular adhesion molecule 1 (ICAM-1) expression and clustering], and favor monocyte adhesion to the endothelium (31). In an *in vitro* model, mTOR inhibition suppressed Ezrin/Radixin/Moesin (ERM) phosphorylation, ICAM-1 clustering and monocyte adhesion to HLA antibody-activated endothelium (32). Another *in vitro* study showed that mTOR inhibition prevented endothelial cell proliferation by downregulating interleukin-8 (IL-8), monocyte chemoattractant protein-1 (MCP-1), transforming growth factor-*β*1 (TGF-*β*1) and von Willebrand factor (vWF) (33). A recently reported cohort study in hypersensitized kidney transplant recipients (calculated panel reactive antibody ≥50%) showed better results in patients treated with EVR vs. MPA, both in combination with CNI and steroids. The risk of BPAR-free survival over 1 year was lower in the EVR group (hazard ratio: 0.32, confidence interval: 0.11–0.90, *P* = 0.031) with numerically lower incidence of dnDSA (4/33 [12.1%] vs. 6/38 [18.2%], *P* = 0.408) (34).

The low number of dnDSAs and events at 12 months in the PP population clearly indicate that close follow-up and monitoring of patients following transplantation play a major role in preventing the development of DSAs. In our study, it is possible that we did not observe any difference in dnDSA development between the 3 immunosuppressive regimens because CNIs were included in all study arms. Moreover, non-adherence increases the likelihood of dnDSA production (1). Similar to other controlled studies, the ATHENA trial conditions were likely to support medication adherence, leading to effective immunosuppression. Although there were high rates of study drug discontinuation and withdrawal in the ATHENA study (7), our primary analysis was based on the PP population. Thus, the treatment effect was estimated among adherent patients, which may have led to the observed comparable rate of dnDSA development across the 3 immunosuppressive regimens. However, it should be noted that similarly low rates of dnDSA development were observed across treatment groups in the ITT population, which included both adherent and non-adherent patients.

There are few limitations which are worth consideration. A follow-up to Month 12 after transplantation does not encompass the long-term effects of the treatment regimens on development of DSA or AMR. Further long-term results, ideally 5- or 10-year data, from prospective studies are required to fully understand the risk of these immunosuppressive regimens on dnDSA and AMR. Nevertheless, late graft loss tends to be related to recurrence of previous disease and adherence to the therapy protocol rather than to the initial immunosuppressive protocol (35). Finally, the substudy was not powered to detect statistical significant differences in clinical outcomes. Thus, the lack of differences in dnDSAs between the groups may be due to the small sample size. The strength of the analysis includes the prospective nature of data sampling, and as such, the data are embedded in a broad analysis of primary and secondary endpoints conducted as part of the primary ATHENA study (7). In the ATHENA study, kidneys were allocated within Eurotransplant according to the standard allocation process (36).

The non-HLA antibodies may also play an important role in transplant rejection and patients with both HLA and non-HLA antibodies have been reported to have poor outcomes and lower graft survival (37–39). A separate substudy of ATHENA evaluated the effect of EVR in combination with CNI on the formation of non-HLA antibodies and graft outcome in KTx patients (40). The results showed that EVR + TAC group had a higher incidence of patients negative for angiotensin II type 1 receptor (AT1R) and endothelin-1 type A receptor (ETAR) antibodies (82.2% and 76.7%, respectively) compared to MPA + TAC group (71.9% and 65.3%, respectively) over Month 12. Similar to findings found for HLA, non-HLA antibodies had no influence on clinical outcomes and no death was reported in patients.

In conclusion, findings from the ATHENA substudy showed that immunosuppression with EVR or MPA in combination with reduced CNI did not increase the risk for dnDSA development. Interestingly, the overall incidence of dnDSA was extremely low and did not directly influence clinical outcome in the first 12 months after KTx. Preformed HLA antibodies (MFI ≥ 500 at baseline, independent of mismatch) and critical HLA antibodies (MFI ≥ 500 at baseline, although being a mismatch) had no influence on clinical outcome, irrespective of EVR or MPA exposure. Clinical outcomes are in line with previous results of ATHENA trial (7).

## Data Availability

Anonymized patient-level data from clinical trials may be shared by Novartis in a consortium called ClinicalStudyDataRequest.com (CSDR) in accordance with Novartis’ policy for sharing clinical trial data. Requests to access the datasets should be directed to https://www.clinicalstudydatarequest.com/Study-Sponsors/Study-Sponsors-Novartis.aspx.
