# Supplementary material for: Everolimus plus reduced calcineurin inhibitor prevents de novo anti-HLA antibodies and humoral rejection in kidney transplant recipients: 12-month results from the ATHENA study
Source: Front Transplant. 2023 Oct 27;2:1264903. doi: 10.3389/frtra.2023.1264903 (PMC11235221; doi:10.3389/frtra.2023.1264903)
Supplement: Supplementary file 1 [file Table1.docx]

Supplementary Material

Everolimus plus reduced calcineurin inhibitor prevents de novo anti-HLA antibodies and humoral rejection in kidney transplant recipients: 12-month results from the ATHENA study

Wolfgang Arns^1*^, Aurélie Philippe^2,3^, Vanessa Ditt^4^, Ingeborg A. Hauser^5^, Friedrich Thaiss^6^, Claudia Sommerer^7^, Barbara Suwelack^8^, Duska Dragun^9^, Jan Hillen^10^, Christiane Schiedel^10^, Anja Elsässer^10^, Björn Nashan^11,12*^

***Corresponding author 1:** Dr. Wolfgang Arns: wolfgang.arns@uni-koeln.de

***Corresponding author 2:** Prof. Björn Nashan: bjoern.nashan@gmail.com

# Supplementary Figures and Tables

## Supplementary Tables

Supplementary Table 1. Baseline demographics and characteristics of patients in ATHENA HLA substudy cohort (ITT population)

| **Variable** | **EVR+TAC (n=206)** | **EVR+CsA (n=198)** | **MPA+TAC (n=202)** | **Total (N=606)** |  |
| --- | --- | --- | --- | --- | --- |
| Age |  |  |  |  |  |
| Mean (SD), years | 54.3 (13.4) | 55.1 (12.7)^a^ | 55.5 (12.1) | 54.9 (12.7)^a^ | 0.665* |
| ≥65 years, n (%) | 53 (25.7) | 59 (29.9)^a^ | 49 (24.3) | 161 (26.6)^a^ |  |
| Male, n (%) | 137 (66.5) | 133 (67.2) | 137 (67.8) | 407 (67.2) | 0.964** |
| White, n (%) | 195 (94.7) | 196 (99.0) | 196 (97.0) | 587 (96.9) | 0.040** |
| Mean BMI (SD), kg/m^2^ | 26.4 (4.3) | 26.6 (4.3) | 26.4 (4.2) | 26.5 (4.2) | 0.880* |
| Panel reactive antibodies, n (%) |  |  |  |  |  |
| 0 | 185 (95.9) | 179 (94.7) | 188 (95.9) | 552 (95.5) |  |
| ≤10 | 7 (3.6) | 7 (3.7) | 6 (3.1) | 20 (3.5) |  |
| >10 and ≤20 | 1 (0.5) | 0 (0.0) | 1 (0.5) | 2 (0.3) |  |
| >20 | 0 (0.0) | 3 (1.6) | 1 (0.5) | 4 (0.7) |  |
| Missing | 13 | 9 | 6 | 28 |  |
| Previous kidney transplant, n (%) | 10 (4.9) | 2 (1.0) | 5 (2.5) | 17 (2.8) |  |
| HLA-A mismatches, n (%) |  |  |  |  |  |
| 0 | 53 (25.7) | 56 (28.3) | 60 (29.7) | 169 (27.9) |  |
| 1 | 102 (49.5) | 91 (46.0) | 94 (46.5) | 287 (47.4) |  |
| 2 | 51 (24.8) | 51 (25.8) | 48 (23.8) | 150 (24.8) |  |
| HLA-B mismatches, n (%) |  |  |  |  |  |
| 0 | 42 (20.4) | 41 (20.7) | 48 (23.8) | 131 (21.6) |  |
| 1 | 92 (44.7) | 80 (40.4) | 75 (37.1) | 247 (40.8) |  |
| 2 | 72 (35.0) | 77 (38.9) | 79 (39.1) | 228 (37.6) |  |
| HLA-DR mismatches, n (%) |  |  |  |  |  |
| 0 | 57 (27.7) | 66 (33.3) | 74 (36.6) | 197 (32.5) |  |
| 1 | 98 (47.6) | 82 (41.4) | 101 (50.0) | 281 (46.4) |  |
| 2 | 51 (24.8) | 50 (25.3) | 27 (13.4) | 128 (21.1) |  |
| Mean cold ischemia time (SD), h | 10.8 (6.3) | 11.7 (6.0) | 11.1 (6.1) | 11.2 (6.1) |  |
| Participant in Eurotransplant Senior Program, n (%) | 23 (11.2) | 23 (11.6) | 22 (10.9) | 68 (11.2) |  |
| Donor characteristics |  |  |  |  |  |
| Mean age (SD), y | 53.4 (15.7) | 56.4 (14.4) | 55.1 (14.8) | 54.9 (15.0) |  |
| Deceased heart-beating, n (%) | 173 (84.0) | 165 (83.3) | 171 (84.7) | 509 (84.0) |  |
| Living-related, n (%) | 23 (11.2) | 22 (11.1) | 22 (10.9) | 67 (11.1) |  |
| Living-unrelated, n (%) | 10 (4.9) | 11 (5.6) | 9 (4.5) | 30 (5.0) |  |

^*^F-test, ^**^Fisher's exact test.

^a^Data from 1 patient missing

Abbreviations: CsA, cyclosporine; EVR, everolimus; HLA, human leukocyte antigen; ITT, intent-to-treat; MPA, mycophenolic acid; SD, standard deviation; TAC, tacrolimus

**Supplementary Table 2.** Patients in ATHENA HLA substudy cohort with HLA antibody data (ITT population)

| **Patient group** | **HLA data at BL, n** | **Preformed HLA at BL,**  **n (%)^a^** | **Preformed critical HLA at BL, n (%)^a^** | **dnDSA,**  **n (%)^b^** |
| --- | --- | --- | --- | --- |
| EVR+TAC (N=206) | 183 | 91 (49.7) | 10 (5.5) | 3 (1.8) |
| EVR+CsA (N=198) | 175 | 80 (45.7) | 12 (6.9) | 11 (7.0) |
| MPA+TAC (N=202) | 177 | 78 (44.1) | 12 (6.8) | 2 (1.3) |
| Total (N=606) | 535 | 249 (46.5) | 34 (6.4) | 16 (3.3) |

Preformed HLA and preformed critical HLA at baseline and DSA detected at MFI ≥500

aPercentage based on number of patients with HLA data at BL; bPercentage based on number of patients with HLA antibodies at BL and post-BL (n=166 in EVR + TAC, 157 in EVR + CsA, and 160 in MPA + TAC groups; total n=483)

BL, baseline; CsA, cyclosporine; dnDSA, de novo donor-specific antibodies; EVR, everolimus; HLA, human leukocyte antigen; ITT, intent to treat; MFI, mean fluorescent intensity; MPA, mycophenolic acid; TAC, tacrolimus

**Supplementary Table 3.** Clinical outcome of patients with preformed HLA and dnDSA (ITT population)

|  | **Clinical event** | **EVR+TAC (N=206)** | **EVR+CsA (N=198)** | **MPA+TAC (N=202)** | **Total (N=606)** |
| --- | --- | --- | --- | --- | --- |
| **Preformed HLA** |  | M=91 | M=80 | M=78 | M=249 |
|  | Overall, n/M (%) | 15/91 (16.5) | 25/80 (31.3) | 5/78 (6.4) | 45/249 (18.0) |
|  | BPAR, n | 9 | 15 | 3 | 27 |
|  | AMR, n | 3 | 3 | 0 | 6 |
|  | Graft loss, n | 4 | 8 | 1 | 13 |
|  | Death, n | 2 | 2 | 1 | 5 |
| **Preformed critical HLA** |  | M=10 | M=12 | M=12 | M=34 |
|  | Overall, n/M (%) | 3/10 (30.0) | 4/12 (33.3) | 0/12 (0) | 7/34 (20.5) |
|  | BPAR, n | 2 | 3 | 0 | 5 |
|  | AMR, n | 0 | 2 | 0 | 2 |
|  | Graft loss, n | 0 | 1 | 0 | 1 |
|  | Death, n | 1 | 0 | 0 | 1 |
| **dnDSA** |  | M=3 | M=11 | M=2 | M=16 |
|  | Overall, n/M (%) | 0/3 (0) | 5/11 (45.4) | 1/2 (50.0) | 6/16 (37.5) |
|  | BPAR, n | 0 | 3 | 1 | 4 |
|  | AMR, n | 0 | 0 | 1 | 1 |
|  | Graft loss, n | 0 | 2 | 0 | 2 |
|  | Death, n | 0 | 0 | 0 | 0 |
| Clinical event defined as biopsy-proven acute rejection, graft loss or death  M, number of paitients with preformed/critical preformed/dnDSA at BL; N, total number of patients; n, number of patients with an event  AMR, antibody-mediated rejection; BL, baseline; BPAR, biopsy-proven acute rejection; CsA, cyclosporine; dnDSA, de novo donor-specific human leukocyte antigen antibodies; EVR, everolimus; HLA, human leukocyte antigen; ITT, intent to treat; MFI, mean fluorescent intensity; MPA, mycophenolic acid; TAC, tacrolimus | | | | | |

## Supplementary Figures

Supplementary Figure 1. ATHENA study design.

*Basiliximab (20 mg) on D0 and D4; #Patients who discontinued study drug returned for assessment at the EOS.

EVR was initiated within 24 h of transplantation and maintained at target trough concentration of 3-8 ng/mL throughout the study period. The target trough concentration of TAC in the EVR+TAC and MPA+TAC arms was 4-8 ng/mL until the end of Month 2 and 3-5 ng/mL thereafter. In the EVR+CsA arm, the target trough concentration of CsA was 75-125 ng/mL until the end of Month 2 and 50-100 ng/mL thereafter. MPA was used either as enteric coated mycophenolate sodium (1.44 g/day) or mycophenolate mofetil (2 g/day). All patients received steroids (≥5 mg/day) until Month 12 (7).

BL, baseline; C0, trough levels; CsA, cyclosporine; D, day; EOS, end of study; EVR, everolimus; KTx, kidney transplant; M, month; MPA, mycophenolic acid; PEoT, Premature End of Treatment; PPW, Premature Patient Withdrawal; RCT, randomized controlled trial; RND, randomization; TAC, tacrolimus.
