# Supplementary figures and images for: Everolimus plus reduced calcineurin inhibitor prevents de novo anti-HLA antibodies and humoral rejection in kidney transplant recipients: 12-month results from the ATHENA study
Source: Front Transplant. 2023 Oct 27;2:1264903. doi: 10.3389/frtra.2023.1264903 (PMC11235221; doi:10.3389/frtra.2023.1264903)

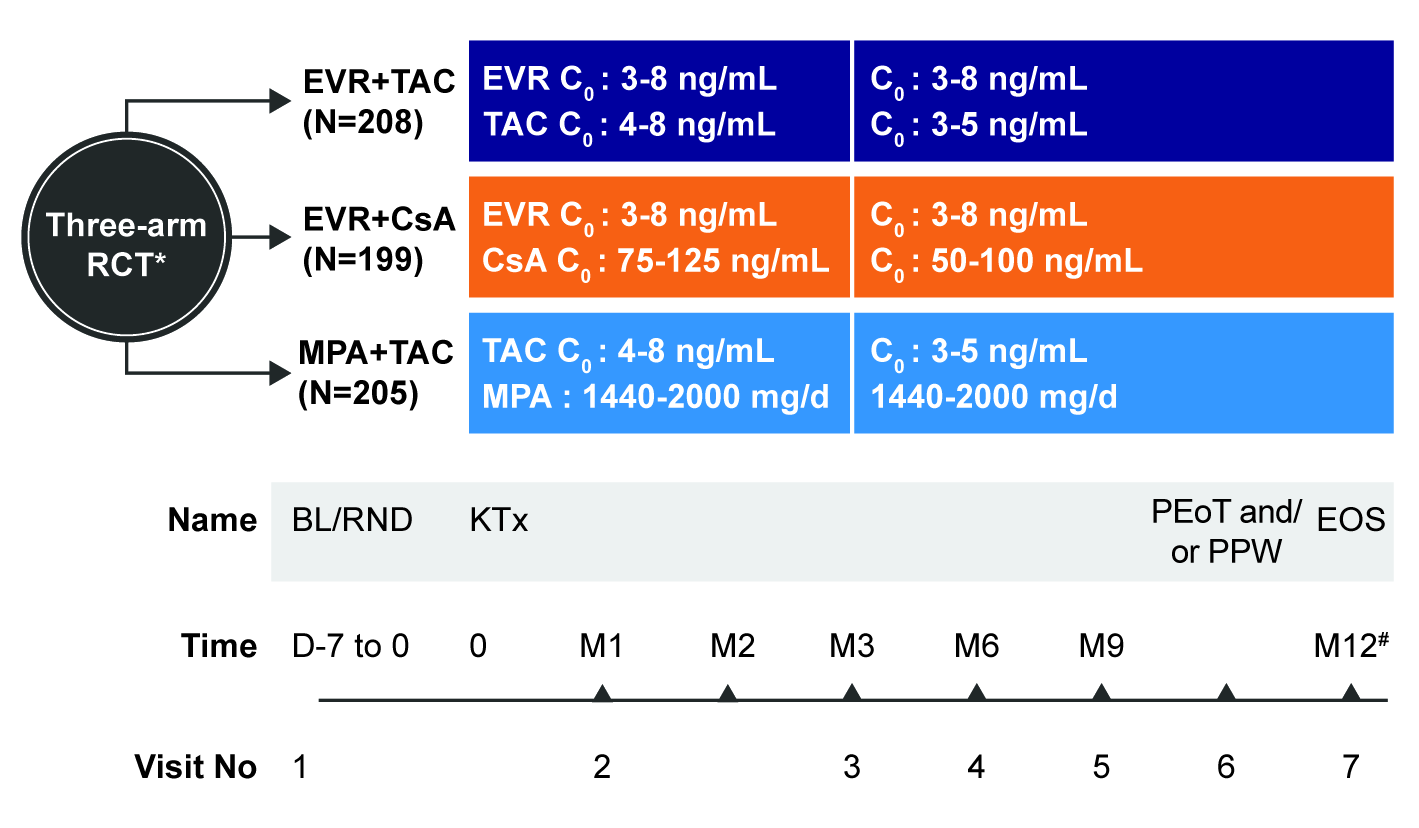

Supplement: Supplementary file 2 [file Image1.tif]
